# Blunt Cerebrovascular Injury in the Elderly With Traumatic Cervical Spine Injuries: Results of a Retrospective Multi-Center Study of 1512 Cases in Japan

**DOI:** 10.1089/neu.2022.0180

**Published:** 2023-06-01

**Authors:** Hidenori Suzuki, Masahiro Funaba, Yasuaki Imajo, Noriaki Yokogawa, Takeshi Sasagawa, Kei Ando, Hiroaki Nakashima, Naoki Segi, Toru Funayama, Fumihiko Eto, Kota Watanabe, Junichi Yamane, Takeo Furuya, Hideaki Nakajima, Tomohiko Hasegawa, Yoshinori Terashima, Shota Ikegami, Gen Inoue, Takashi Kaito, Satoshi Kato

**Affiliations:** ^1^ Department of Orthopedics Surgery, Yamaguchi University Graduate School of Medicine, Yamaguchi, Japan.; ^2^ Department of Orthopedic Surgery, Graduate School of Medical Sciences, Kanazawa University, Ishikawa, Japan.; ^3^ Department of Orthopedics Surgery, Toyama Prefectural Central Hospital, Toyama, Japan.; ^4^ Department of Orthopedic Surgery, Graduate School of Medicine, Nagoya University, Nagoya, Japan.; ^5^ Department of Orthopedic Surgery, Faculty of Medicine, University of Tsukuba, Ibaraki, Japan.; ^6^ Graduate School of Comprehensive Human Sciences, University of Tsukuba, Ibaraki, Japan.; ^7^ Department of Orthopedic Surgery, Keio University School of Medicine, Tokyo, Japan.; ^8^ Department of Orthopedic Surgery, National Hospital Organization Murayama Medical Center, Tokyo, Japan.; ^9^ Department of Orthopedic Surgery, Graduate school of Medicine, Chiba University, Chiba, Japan.; ^10^ Department of Orthopedics and Rehabilitation Medicine, Faculty of Medical Sciences, University of Fukui, Fukui, Japan.; ^11^ Department of Orthopedic Surgery, Hamamatsu University School of Medicine, Shizuoka, Japan.; ^12^ Department of Orthopedic Surgery, Sapporo Medical University, Sapporo, Japan.; ^13^ Department of Orthopedic Surgery, Matsuda Orthopedic Memorial Hospital, Sapporo, Japan.; ^14^ Department of Orthopedic Surgery, Shinshu University School of Medicine, Nagano, Japan.; ^15^ Department of Orthopedic Surgery, Kitasato University School of Medicine, Kanagawa, Japan.; ^16^ Department of Orthopedic Surgery, Osaka University Graduate School of Medicine, Osaka, Japan.

**Keywords:** blunt cerebrovascular injury, cervical spinal cord injury, cervical spine fractures, elderly patients, multi-center study

## Abstract

This study is nationwide retrospective multi-center study to investigate the incidence and characteristics of blunt cerebrovascular injury (BCVI) in elderly Japanese patients with traumatic cervical spine injuries (CSI) including spinal cord injury (SCI) without major bone injury. The study enrolled 1512 patients (average age: 75.8 ± 6.9 years; 1007 males, 505 females) from 33 nationwide institutions, and 391 (26%) of the participants had digital subtraction angiography and/or computed tomography angiography. Fifty-three patients were diagnosed as having BCVI by angiography. We assessed neurological evaluation, comorbidities and classification of CSI in the elderly patients with/without BCVI and collected 6-month follow-up data on treatment, complications, and patient outcome. We also statistically analyzed the relative risk (RR) and relationship between BCVI and other factors. Significant differences were identified between BCVI (+) (*n* = 53) and (–) (*n* = 1459) patients with American Spinal Injury Association Impairment Scale (ASIA) A, C, D, cervical fracture, C3-7 injury level (AO type F and/or C), cervical dislocation, spinal surgery for CSI, tetraplegia type of SCI, and/or head injury. Fifty-three (3.5%) elderly patients had CSI complicated by BCVI including 10 (19%) cases of Denver grade I, four (7%) of grade II, 1 (2%) of grade III, 29 (55%) of grade IV, and nine (17%) of grade V. Sixteen cases were treated by interventional radiology. Rates of mortality and brain infarction from BCVI were 0.13% and 0.40%, respectively. RR of BCVI was significantly higher in the elderly cervical injury patients with head injury, severe neurological deficit, ASIA A (RR: 4.33), cervical fracture at the C3-7 level (RR: 7.39), and cervical dislocation at the C1-6 level (RR: 3.06-7.18). In conclusion, 53 (3.5%) elderly patients were complicated with BCVI. BCVI more frequently complicated head injury, severe neurological deficit (ASIA A or tetraplegia), AO type F, and/or C fractures and cervical dislocation in these patients. Six patients (11%) suffered brain infarction and two patients died from BCVI.

## Introduction

Blunt cerebrovascular injury (BCVI) of carotid or vertebral vessels is diagnosed in about one in 1000 (0.1%) hospitalized trauma patients in the United States unless a screening program has been started.^1^ Unfortunately, most of these injuries are diagnosed after symptoms develop secondarily to central nervous system ischemia, with resulting rates of neurologic morbidity of nearly 80% and associated mortality of almost 40%.^2^ An association between cervical spine injuries (CSI) and BCVI, especially vertebral artery (VA) injury (VAI), was reported, and >70% of the patients with VAI have CSI or spinal cord injury (SCI).^3–7^

The aging of the Japanese society as a globally occurring phenomenon is affecting the epidemiology of traumatic CSI, with the prevalence of SCI increasing in older adults.^8–15^ The injury characteristics, cause of trauma, complications following injury, symptom progress, neurological recovery, outcome, treatment approach, and BCVI-related data in elderly patients are different from those of younger and middle-age patients.^6,7,8–13,15^ Despite a consistent trend toward older age when CSI occurs, only a few articles have reported on the most severe complication caused by CSI in elderly patients, that of BCVI.^6,7,15^ When the doctors miss several important diagnostic clues, brain dysfunction or fatal complications will be led by medical misdiagnosis. Therefore, diagnosis of BCVI at the first emergency unit is quite important. The greatest limitation of previous studies in elderly CSI was the small number of participants. Although this multi-center large cohort study is retrospective in nature, we evaluated the largest number of older individuals (aged ≥65 years) with CSI with BCVI; therefore, we provide stronger evidence than previous reports in elderly BCVI with CSI in this study.^16^

CSI with concomitant neurologic injury is associated with a mortality rate of up to 50% in the elderly.^15–21^ Even though only a small number of patients present with neurologic deficits following acute traumatic VAI, consequences can be severe if an ischemic or embolic phenomenon occurs after delayed presentation due to a missed diagnosis.^6,7^ It is thus critical to recognize BCVI early in this vulnerable population. Screening guidelines are available to identify patients at high risk for BCVI following CSI to determine those who should undergo cervical computed tomography angiography (CTA).^6,7^ Still, patients presenting with atypical radiographic or injury findings may be missed, and contrast media injected during CTA may harm kidney function, especially in elderly patients. Therefore, characteristics of the risk factors for BCVI in the elderly should be revealed, and a routine screening system for elderly patients with CSI is needed to prevent severe complications following BCVI.^6,7,15^

Therefore, the purposes of our study were to determine the incidence of BCVI and real clinical outcomes in elderly Japanese people following BCVI caused by CSI not defined by the newly classified high-risk patterns of injury through analysis of elderly CSI patients in a large nationwide database and to clarify the characteristics of risk factors of BCVI that lead to severe complications including mortality and brain infarction in these patients.^6,7,16^

## Methods

### Study participants

We reviewed a multi-center database of 1524 consecutive patients age ≥65 years with CSI, including SCI without major bone injury, in 33 domestic institutions between 2010 and 2020, which was compiled by JASA (Japan Association of Spine Surgeons with Ambition). Twelve patients with cervical metastasis and/or multiple missing data were excluded. Therefore, we finally reviewed the data for 1512 patients in this study. The study design was approved by the institutional review board at each participating hospital and by the Institutional Review Boards of Yamaguchi University (2020-133). Informed consent was obtained via an opt-out system at each institution. Patients who declined participation were excluded. The study design was in compliance with the Declaration of Helsinki.

Patients age 65 years or older with traumatic CSI who were treated conservatively or surgically from 2010 to 2020 at an institution registered with JASA were included. In total, 1512 patients from 33 nationwide institutions were enrolled in this study (average age: 75.8 ± 6.9 years; 1007 males, 505 females).^16,17^ Data including age at injury, sex, height and weight at first visit, past/present cigarette smoking, spinal surgery for the CSI, and comorbidities (hypertension, cerebrovascular disease, cardiovascular disease, ossification of posterior longitudinal ligament [OPLL] and head injury) were collected. Characteristics of the study population are shown in Table 1.

**Table 1.** Characteristics of the Study Population

	Overall (*n* = 1512)	BCVI - (*n* = 1459)	BCVI + (*n* = 53 )	*p* Value
Ages (years) ± SD	75.8 ± 6.9	75.8 ± 7.0	74.5 ± 5.9	n.s.
Sex (male/female)	1007/505	971/488	36/17	n.s.
Height (cm) ± SD	160 ± 8.8	159 ± 9.2	160 ± 8.4	n.s.
Weight (kg) ± SD	58.2 ± 11	56.0 ± 11	58.5 ± 11	n.s.
Presence of pre-injury medical comorbidity of hypertension	731 (48.3%)	706 (48.4%)	25 (47.1%)	n.s.
Past/present cigarette smoker	288 (19.0%)	280 (19.1%)	8 (15.1%)	n.s.
Presence of pre-injury medical comorbidity of cerebrovascular disease	145 (9.6%)	142 (9.7%)	3 (5.7%)	n.s.
Presence of pre-injury medical comorbidity of cardiovascular disease	134 (8.9%)	131 (9.0%)	3 (5.7%)	n.s.
Comorbidity of OPLL	332 (21.9%)	319 (21.9%)	13 (24.5%)	n.s.
Comorbidity of head injury	217 (14.3%)	199 (13.6%)	18 (34.0%)	<0.001
Spinal surgery for cervical fracture/dislocation	903 (59.7%)	858 (58.8%)	45 (84.9%)	<0.001

The *p* values of differences between the BCVI + and − groups were calculated by Welch's t-test or Fisher's exact test.

BCV, blunt cerebrovascular injury; SD, standard deviation; n.s., not significant; OPLL, ossification of posterior longitudinal ligament.

### Neurological evaluation and classification of cervical spine fracture/dislocation

Neurological disability was assessed as a parameter of neurological impairment using the American Spinal Injury Association Impairment Scale (ASIA; Grade A: complete impairment to Grade E: normal function).^2,5,21-23^ We also assessed the type of SCI (none, type of central cord syndrome and tetraplegia) by magnetic resonance imaging and neurological symptoms of the upper and lower limbs and trunk (Table 2).^2,5,21-23^

**Table 2.** Characteristics of the Elderly Patients with Cervical Fracture/Dislocation and SCI

	Overall (*n* = 1512)	BCVI - (*n* = 1459)	BCVI + (*n* = 53)	*p* Value
ASIA				
A	125	108 (7.4%)	17 (32.1%)	<0.001
B	68	65 (4.5)	3 (5.7)	n.s.
C	336	330 (22.6)	6 (11.3)	0.052
D	518	510 (35.0)	8 (11.3)	0.028
E	465	446 (30.6)	19 (35.8)	n.s.
Number of cervical fractures	834	787 (53.9%)	47 (88.7%)	<0.001
Injury level (There is some overlap)				
C1	51	48 (3.3)	3 (5.7)	n.s.
C2	315	303 (20.8)	12 (22.6)	n.s.
C3-7	556	513 (35.2)	43 (81.1)	<0.001
Number of cervical dislocations	228	206 (14.1%)	22 (41.5%)	<0.001
C1/2	20	18 (0.9)	2 (3.8)	0.153
C2/3	9	8 (0.5)	1 (1.9)	0.275
C3/4	8	7 (0.5)	1 (1.9)	0.249
C4/5	29	23 (1.6)	6 (11.3)	<0.001
C5/6	62	54 (3.7)	8 (15.1)	<0.001
C6/7	69	65 (4.5)	4 (7.5)	n.s.
C7/T1	11	11 (0.8)	0	n.s.
Type of SCI				
None	457	440 (30.2%)	17 (32.1%)	n.s.
Central cord syndrome	542	534 (36.6)	8 (15.1)	0.001
Tetraplegia	511	485 (35.0)	26 (49.1)	0.017
Unknown	2	0	2 (3.8)	0.001

The *p* values of differences between the BCVI + and - groups were calculated by χ2 test or Fisher's exact test, where appropriate.

SCI, spinal cord injury; BCVI, blunt cerebrovascular injury; ASIA, American Spinal Cord Injury Association; n.s., not significant

Collected radiographic data included images of the cervical fracture, CSI findings (level of fracture and articular facet dislocation, classified as upper cervical spine [C1-C2] fracture, C3 or lower injury), AO classifications, and OPLL as detected by plain radiography and/or computed tomography (CT; Table 2 and Table 4).^21–24^ Based on AO classification, we classified as follows: AO: A, vertebral compression injuries; AO: B, vertebral tension band injuries; C, vertebral translation injury; AO: F, facet injuries; and AO: BL, bilateral facet injuries. In addition, we defined the combination fractures that, for example, AO: C-BL mean vertebral translation injury combined with facet injuries etc. (Table 4).^24^

**Table 4.** Incidence and Relative Risk of Each Category of Injury in the 53 Elderly Patients with BCVI

	RR	95% CI	*p* Value	BCVI (*n* = 53)* n *(%)	No BCVI (*n* = 1459)* n *(%)
Head injury	**2.49**	1.67-3.70	<0.001	18 (34.0)	199 (13.6)
ASIA					
A	**4.33**	2.81-6.67	<0.001	17 (32.1)	108 (7.4)
B	1.27	0.41-3.91	0.677	3 (5.7)	65 (4.5)
C	0.50	0.19-1.04	0.052	6 (53.9)	330 (22.6)
D	0.43	0.23-0.82	0.3	8 (11.3)	510 (35.0)
E	1.17	0.81-1.69	0.003	19 (35.8)	446 (30.6)
Cervical fracture	**1.64**	1.48-1.83	0.003	47 (88.7)	787 (53.9)
Injury level					
C1	1.27	0.41-3.91	0.677	3 (5.7)	48 (3.3)
C2	1.09	0.66-1.81	0.741	303 (20.8)	12 (22.6)
C3-7	**7.39**	3.75-14.60	<0.001	43 (81.1)	513 (35.2)
Cervical dislocation	**2.94**	2.08-4.15	<0.001	22 (41.5)	206 (14.1)
Dislocation level					
C1/2	3.06	0.73-12.84	0.112	2 (3.8)	18 (0.9)
C2/3	3.44	0.44-27.02	0.213	1 (1.9)	8 (0.5)
C3/4	3.93	0.49-31.39	0.165	1 (1.9)	7 (0.5)
C4/5	**7.18**	3.05-16.90	<0.001	6 (11.3)	23 (1.6)
C5/6	**4.07**	2.05-8.13	<0.001	8 (15.1)	54 (3.7)
C6/7	1.69	0.64-4.47	0.289	4 (7.5)	65 (4.5)
AO classification (C3-7)					
A	1.05	0.47-2.37	0.898	7 (13)	184 (13)
A-F	**2.97**	0.67-13.1	0.131	2 (3.8)	19 (1.3)
B	0.55	0.19-1.60	0.043	4 (7.5)	44 (3.0)
B-F	**2.69**	0.81-8.92	<0.001	4 (7.5)	10 (0.7)
BL	—	—	—	1 (1.9)	0
C	**1.85**	0.71-4.79	0.004	6 (11)	22 (1.5)
C-BL	**1.77**	0.56-5.55	<0.001	4 (7.5)	15 (1.0)
C-F	**9.44**	4.56-19.5	<0.001	19 (36)	19 (1.3)
F	**6.54**	2.40-17.8	<0.001	8 (15)	9 (0.6)
Type of SCI					
None	1.06	0.71-1.59	0.765	17 (32.1)	440 (30.2)
Central cord syndrome	**0.41**	0.21-0.78	0.001	8 (15.1)	534 (36.6)
Tetraplegia	**1.48**	1.11-1.96	0.017	26 (49.1)	485 (35.0)
Comorbidity of OPLL	1.12	0.69-1.81	0.645	13 (24.5)	319 (21.9)
Comorbidity of hypertension	0.93	0.69-1.26	0.649	24 (45.3)	707 (48.5)

Bold indicates that RR is high.

RR, relative risk; CI, confidence interval; BCVI, blunt cerebrovascular injury; ASIA, American Spinal Cord Injury Association; SCI, spinal cord injury; OPLL, ossification of posterior longitudinal ligament; AO classification: A, vertebral morphology/compression injuries; B, vertebral morphology/tensionband injuries; C, vertebral morphology/translation injury; F, facet injuries; BL, bilateral facet injuries.

### Diagnostics, classification of BCVI, and treatment

To diagnose BCVI, digital subtraction angiography (DSA) and/or CTA of the carotid and vertebral arteries was performed by neuroradiologists. Signs/symptom for DSA and/or CTA indication are listed below: 1) potential arterial hemorrhage from neck/nose/mouth; 2) expanding cervical hematoma; 3) focal neurologic defect: transit ischemic attack, hemiparesis, vertebrobasilar symptoms, Horner's syndrome; 4) stroke on CT or magnetic resonance imaging; 5) severe neurologic deficit; 6) high energy transfer mechanism (complicated with blunt cardiac rupture or thoracic vascular injuries; 7) complex skull fracture/basilar skull fracture/occipital condyle fracture; 8) severe traumatic brain injury with Glasgow coma scale (GCS) <6; and 9) unstable cervical spine fracture or dislocation.^6,7^

None of the study patients had a carotid injury. We classified all identified BCVI types by Denver grading scale (Table 3).^6,7^ Follow-up data of 51 of the 53 patients with BCVI were available for 6 months after injury except for two patients dead due to complications of BCVI. We also collected the 6-month follow-up data on treatment, complications, and outcome in the patients with BCVI and statistically analyzed the relative risk (RR) and relationship between BCVI and the other factors. 

**Table 3.** Denver Grading Scale in BCVI, Treatment and Outcome

	Treatment (%)			Complications/outcome (%)		
	None	Medication	Vascular IVR	No complications	Brain infarction	Dead
Overall (*n* = 53)	35 (66)	1 (2)	17 (32)	45 (85)	6 (11)	2 (4)
Grade I (*n* = 10)	8 (80)	0	2 (20)	10 (100)	0	0
II (*n* = 4)	3 (75)	0	1 (25)	3 (75)	1 (25)	0
III (*n* = 1)	0	0	1 (100)	1 (100)	0	0
IV (*n* = 29)	24 (83)	1 (3)	4 (14)	23 (79)	4 (14)	2 (7)
V (*n* = 9)	0	0	9 (100)	8 (89)	1 (11)	0

BCVI, blunt cerebrovascular injury; IVR, interventional radiology; VA, vertebral artery;

IMH, intramural hematoma; AVF, arteriovenous fistula.

*Grade I: Vessel wall irregularity, dissection, or IMH with <25%, luminal stenosis*

*II: Any raised intimal flap, Any intraluminal thrombus, Dissection or IMH with >25% luminal stenosis*

*III: Arterial pseudoaneurysm*

*IV: Arterial occlusion*

*V: Arterial transection and/or AVF*

### Statistical analysis

Data are presented as the mean ± standard deviation. In the analysis of BCVI versus non-BCVI, the Welch's t-test was used to evaluate continuous variables and the Chi-square test (χ2 test) and Fisher's exact test to evaluate categorical variables (Table 1 and Table 2). The relative risk (RR) of BCVI and 95% confidence intervals (CI) of the dependent factors were calculated (Table 4). Statistical significance was set at *p* < 0.05. All analyses were performed with StatFlex Ver. 7 for Windows (Artec, Osaka, Japan). 

## Results

### Baseline characteristics

In total, 1512 elderly patients with traumatic CSI including SCI without major bone injuries were identified in our database during the study period (Table 1). In the database, 391 (26%) of the 1512 patients underwent DSA and/or CTA of the carotid and vertebral arteries, and 53 patients were diagnosed as having BCVI. Mean ages were 75.8 ± 6.9 years for all patients, 74.5 ± 5.9 years for those with BCVI, and 75.8 ± 7.0 years for those without BCVI (Table 2). Two-thirds of the BCVI (+) and BCVI (–) patients were men. Cervical bone injuries (fractures and/or dislocations) were found in 59% of the patients, approximately half of whom had associated SCI, and 41% had CSI without bony injuries. Characteristics of the study population are shown in Tables 1 and 2.

A significant difference between BCVI (+) and BCVI (–) was identified in patients with ASIA A, C, D, cervical fracture, C3-7 injury level, cervical dislocation, spinal surgery for cervical fracture/dislocation, type of SCI and/or head injury (Table 1 and Table 2).

### Frequency of BCVI in elderly patients with CSI and/or SCI

Fifty-three (3.5%) elderly patients with CSI complicated with BCVI, of whom 10 (19%) cases of Denver grade I, four (7%) of grade II, one (2%) of grade III, 29 (55%) of grade IV, and nine (17%) of grade V (Table 3). Denver grade scale in BCVI was classified as follows: grade I: vessel wall irregularity, dissection, or IMH with <2 5%, luminal stenosis; grade II: any raised intimal flap, any intraluminal thrombus, dissection or IMH with >25% luminal stenosis; grade III: arterial pseudoaneurysm; grade IV: arterial occlusion; and grade V: arterial transection and/or AVF.^6,7^

### Frequency of DSA and/or CTA of the carotid and vertebral arteries in elderly patients with CSI

In our institutions, 391 (26%) patients underwent early angiography to diagnose BCVI. 14 % of patients following DSA and/or CTA were diagnosed BCVI. In 6 months following CSI, eight patients had complicated with delayed brain infarction after CSI. Two patients who underwent CTA just after CSI, however, did not complicate with BCVI. In addition, six patients did not undergo DSA and/or CTA because they did not have the symptoms of criteria in BCVI described in previous session.^6,7^ Unfortunately, the cause of the brain infarction remained unknown.

### Treatment, complications, and outcome for each BCVI type

Among the 53 patients with BCVI, treatment was not focused on BCVI in 36 (68%), one (2%) patient was treated by medication using anticoagulants, and 16 (30%) were treated by interventional radiology (IVR). Among the patients with VA occlusion, dissection or vasoconstriction, 78-100% received conservative treatment. In contrast, all (100%) patients with VA rupture underwent vascular IVR treatment (Table 3).

Forty-five patients (85%) had no complications following treatment; however, dix patients (11%) suffered complications of brain infarction caused by BCVI. Unfortunately, two of the BCVI (+) patients died due to BCVI (Table 3). Five (14%) of 36 patients without treatment of BCVI and two (13%) of 16 patients treated by IVR had complicated brain infarction. 

In summary, mortality and brain infarction rates in the elderly Japanese patients with CSI complicated by BCVI were 0.13% and 0.40%, respectively.

### Incidence and relative risk for each risk factor for patients with BCVI (Table 3) 

We calculated the relative risk (RR) for each risk factor and show them in Table 4. In summary, BCVI more frequently complicated head injury, severe neurological deficit (ASIA A and SCI of tetraplegia), cervical fracture (AO type C and/or F and their combination), and that at the C3-7 level and cervical dislocation and that at the C1-6 level in the elderly patients with CSI. However, the patients without severe neurological deficit or SCI type of central cord syndrome were rarely complicated with BCVI.

## Discussion

After a traumatic injury, compared with younger patients, elderly patients experience higher mortality rates and more severe neurological deficit, even if the severity of the injury is similar, and multi-factorial causes such as deterioration of spinal cord function, poor cardiopulmonary reserve, numerous medications, and a greater number of medical comorbidities can result in poor outcomes.^15–23,25–28^ In addition, the mechanism of cervical injury in elderly patients differs from that in younger patients in that even low impact mechanisms can cause moderate to severe injuries to the spine and spinal cord in the elderly.^15-17,25–28^ In the present study, we revealed specific characteristics, frequency, outcome and risk factors in elderly cervical injury patients with BCVI collected from a nationwide retrospective database in Japan and discuss the differences from previous reports on common BCVI.

BCVI is associated with complex CSI and SCI, but the diagnosis of stroke after BCVI can be difficult because most strokes occur after an asymptomatic lag time of hours to weeks.^6,7^ However, the guidelines for BCVI may not catch patients presenting with atypical radiographic or injury findings.^6,7^ Early diagnosis provides a narrow therapeutic window that can help prevent the development of thromboembolic complications and adverse outcomes.^6,7^ To ensure an early diagnosis, we need to identify the risk factors that correlate with BCVI when the patients initially arrive at the emergency department.

In our analysis, 53 (3.5%) elderly patients with CSI were complicated with BCVI. Several articles have suggested that among all blunt trauma patients, the prevalence of BCVI is around 1.4-1.6%.^6,7,29-31^ It was reported that VAI occurred in 25% of patients with significant fractures including subluxation and extension of fractures into the foramen transversarium, facet, spinous process, or vertebral body.^6,7^ It is hard to determine whether the 3.5% occurrence rate of BCVI in the elderly cervical injury patients in the present study is a lower frequency or not, but several reports have noted that when cervical fractures extend into thoracic fractures or distraction/flexion injuries, the incidence of VAI is high, at 8% to 75%.^6,7,29-31^ The Denver criteria, a BCVI screening tool, state that routine evaluation for BCVI is only indicated when a high-risk cervical spine injury pattern is present (i.e., subluxation, fracture extension into the transverse foramen, or fractures within the upper cervical spine [C1–C3]).^6,7^

In the present study, we revealed the risk factors and RR for elderly patients with CSI complicated with BCVI. BCVI was a more frequent complication with head injury (RR: 2.49), severe neurological deficit [ASIA A (RR: 4.33), cervical fracture and that at the C3-7 level (RR: 7.39), AO type: A-F (RR: 2.97), B-F (RR: 2.69), C (RR: 1.85), C-BL (RR: 1.77), C-F (RR: 9.44), F (RR: 6.54)]; and cervical dislocation (RR: 2.94) that were at the C1-6 level (RR: 3.06-7.18) in the elderly patients with CSI (Table 4). These RRs indicate that we should use DSA and/or CTA to examine the carotid arteries and VAs to diagnose BCVI when elderly patients have these comorbidities combined with spinal injury.

Previous articles have indicated the risk factors of BCVI to be cervical spine injury, spinal dislocation, petrous bone fracture, diffuse axonal injury, Lefort II or III fractures, traumatic intracranial hemorrhage, and Glasgow Coma Scale score ≤6.31. Another article reported the incidence of BCVI in elderly ground-level falls was 0.15%, and higher risk factors were any cervical spine injury, basilar skull fracture and low Glasgow Coma Scale score at admission.^6,7,32^ Several papers have reported the mechanism and characteristics of VAI, which commonly occurs in the V2 or V3 segments where the VAs pass through the transverse foramina or the windows around C1.^6,7^ These articles also reported that other mechanisms include direct injury resulting from associated vertebral fractures involving the transverse foramen and hyperextension or stretch injury caused by tethering the artery within the lateral masses of the cervical spine.^6,7^ Several authors reported the middle cervical spine to be the most affected fracture level in patients with VAI.^6,7,29-31^

We think that the common mechanism and risk factors in the elderly patients in the present study were a little different than those of common BCVI because low energy trauma mostly caused CSI and SCI in elderly patients. In addition, the occurrence rate was lower than those in previous reports on BCVI. Our data showed that 304 (20%) of the 1512 patients 65 years or older with SCI had no major bone injury and that SCI in 20% of the patients was caused by low-energy trauma.^16,17^ Therefore, we think that the occurrence rate of 3.5% in the elderly patients was lower than that typically seen in young and adult patients. Previous articles have not reported the risk factors based on AO classifications. ^6,7,24^ In this study, we newly revealed that AO type: F (facet injuries), BL (bilateral facet injuries), C (vertebral translation injury), and their combination were high risk factors in BCVI complications in elderly CSI patients. Our data suggested that translational dislocated fractures, facet fractures, and dislocation cervical spine trauma mostly caused the BCVI; in addition, more severe combination trauma were higher risk factors in elderly BCVI even if these were caused by low energy trauma.

Treatments and outcomes in common BCVI patients were reported in several articles.^6,7,29-31^ Over 50% of Denver grade I (intimal irregularity and <25% luminal stenosis on angiography) patients experienced complete healing with anticoagulation therapy and required no further treatment, whereas in over 40% of patients with Grade II (intimal irregularity and >25% luminal stenosis in angiography), injuries progressed to pseudoaneurysm formation.^6,7,29-31^ The choice of endovascular treatment is guided by injury extent and the clinical picture of the patient's symptoms.^6,7^ Treatment by vessel sacrifice may be reasonable in dissections with distal emboli and good collateral flow, but stenting may be preferrable if collateral flow is poor or bilateral vessel injury is present.^6,7^ The natural history of traumatic VAI shows that greater than 90% of stenotic dissections resolve within several months, and more than half of all occluded vessels recanalize. ^6,7,29-31^

Treatments and outcomes at 6 months of follow-up in the 53 elderly patients with BCVI are shown in Table 3. Only patients with VAI were included in the present study and not those with carotid vessel injury. We think the reason for no carotid vessel injury in this series is that elderly CSI patients are mostly caused by low energy trauma because most of carotid vessel injury is complicated following high energy multiple trauma.^7^

The patients suffered VA occlusion, VA dissection, VA rupture, and vasoconstriction that we classified using the Denver grading scale. In the majority of patients, treatment was not focused on VAI, only one patient was treated by anticoagulants, and 16 patients were treated by IVR. Unfortunately, brain infarction occurred in six patients, and two patients died due to VAI (Table 3). In this study, the mortality rate in the elderly patients with CSI caused by VAI was 0.13% and that of brain infarction was 0.40%. Previous reports revealed the complication rate of brain infarction to be 7.1-33.3% following VAI. ^6,7,29-31^ To prevent subsequent stroke in patients with traumatic VAI, anticoagulant or antiplatelet therapy is recommended.^6,7^ Due to the current lack of sufficient evidence, the effectiveness of endovascular surgery to reduce the risk of stroke in these patients remains unclear. ^6,7,29-31^ Overall, the treatments for each type of BCVI are still undefined and controversial.

We think that the present complication and treatment rates of VAI were lower than those of previous reports on elderly patients. In our institutions, 26% of the patients underwent early angiography to diagnose BCVI, and this may have led to a lower complication rate for BCVI. In six months following CSI, eight patients had complicated with delayed brain infarction after CSI. Two patients who underwent CTA just after CSI, however, did not complicate with BCVI. In addition, six patients did not undergo DSA and/or CTA because they did not have the symptoms of criteria in BCVI. The causes of delayed brain infarction are still unknown; however, all of the eight patients had comorbidities of hypertension, cerebrovascular disease and/or cardiovascular disease. In addition, Trauma type of AO were type A1 or A2 stable type fractures or SCI without major bone injury in these patients. From our previous results, we think that the cause of delayed infarction was not traumatic CSI.

## Conclusions

We investigated the characteristics and incidence of BCVI in elderly patients with CSI in Japan collected from a nationwide database. Fifty-three (3.5%) elderly patients suffered BCVI, which was a more frequent following complications. In elderly CSI patients, we suggest that BCVI screening is necessary in the early stage for the patients with head injury, severe neurological deficit (ASIA A SCI and tetraplegia type), cervical fracture AO type F (facet injuries), BL (bilateral facet injuries), C (vertebral translation injury), and their combination at the C3-7 level and cervical dislocation at the C1-6 level, even if they were caused by low energy trauma and not multiple trauma. In addition, past/present cigarette smoking and comorbidities (hypertension, cerebrovascular disease, cardiovascular disease, OPLL) are not the risk factors of BCVI in elderly CSI patients. In this study, six of CSI patients with BCVI (11%) suffered brain infarction caused by BCVI, and unfortunately, two patients died from BCVI.

## JASA Study Group 

Koji Akeda, Department of Orthopedic Surgery, Mie University Graduate School of Medicine, Mie, Japan; Haruki Funao, Department of Orthopedic Surgery, School of Medicine, International University of Health and Welfare, Chiba, Japan, Department of Orthopedic Surgery, International University of Health and Welfare Narita Hospital, Chiba, Japan, and Department of Orthopedic Surgery and Spine and Spinal Cord Center, International University of Health and Welfare Mita Hospital, Tokyo, Japan; Ko Hashimoto, Department of Orthopedic Surgery, Tohoku University Graduate School of Medicine, Miyagi, Japan; Yohei Haruta, Department of Orthopedic Surgery, Graduate School of Medical Sciences, Kyushu University, Fukuoka, Japan; Ryosuke Hirota, Department of Orthopedic Surgery, Sapporo Medical University, Sapporo, Japan; Yoichi Iizuka, Department of Orthopedic Surgery, Gunma University, Graduate School of Medicine, Gunma, Japan; Shiro Imagama, Department of Orthopedic Surgery, Nagoya University Graduate School of Medicine, Nagoya, Japan; Masayuki Ishihara, Department of Orthopedic Surgery, Kansai Medical University Hospital, Osaka, Japan; Yuji Kakiuchi, Department of Orthopedic Surgery, Kobe University Graduate School of Medicine, Kobe, Japan; Kenichiro Kakutani, Department of Orthopedic Surgery, Kobe University Graduate School of Medicine, Kobe, Japan; Kenji Kato, Department of Orthopedic Surgery, Nagoya City University Graduate School of Medical Sciences, Nagoya, Japan; Kenichi Kawaguchi, Department of Orthopedic Surgery, Graduate School of Medical Sciences, Kyushu University, Fukuoka, Japan; Katsuhito Kiyasu, Department of Orthopedic Surgery, Kochi Medical School, Kochi University, Kochi, Japan; Kosuke Misaki, Department of Orthopedics, Traumatology and Spine Surgery, Kawasaki Medical School, Okayama, Japan; Masashi Miyazaki, Department of Orthopedic Surgery, Faculty of Medicine, Oita University, Oita, Japan; Kazuo Nakanishi, Department of Orthopedics, Traumatology and Spine Surgery, Kawasaki Medical School, Okayama, Japan; Tetsuro Ohba, Department of Orthopedic Surgery, University of Yamanashi, Yamanashi, Japan; Seiji Okada, Department of Orthopedic Surgery, Osaka University Graduate School of Medicine, Osaka, Japan; Yoshito Onoda, Department of Orthopedic Surgery, Tohoku University Graduate School of Medicine, Miyagi, Japan; Yasushi Oshima, Department of Orthopedic Surgery, The University of Tokyo Hospital, Tokyo, Japan; Bungo

Otsuki, Department of Orthopedic Surgery, Graduate School of Medicine, Kyoto University, Kyoto, Japan; Daisuke Sakai, Department of Orthopedics Surgery, Surgical Science, Tokai University School of Medicine, Kanagawa, Japan; Takuya Sakamoto, Department of Orthopedics Surgery, Yamaguchi University Graduate School of Medicine, Yamaguchi, Japan; Munehiro Sakata, Department of Orthopedics, Graduate School of Medical Science, Kyoto Prefectural University of Medicine, Kyoto, Japan and Department of Orthopedics, Saiseikai Shiga Hospital, Shiga, Japan; Munehiro Sakata, Department of Orthopedics, Graduate School of Medical Science, Kyoto Prefectural University of Medicine, Kyoto, Japan and Department of Orthopedics, Saiseikai Shiga Hospital, Shiga, Japan; Hirokatsu Sawada, Department of Orthopedic Surgery, Nihon University School of Medicine, Tokyo, Japan; Shoji Seki, Department of Orthopedic Surgery, Faculty of Medicine, University of Toyama, Toyama, Japan; Eiki Shirasawa, Department of Orthopedic Surgery, Kitasato University School of Medicine, Kanagawa, Japan; Nobuyuki Suzuki, Department of Orthopedic Surgery, Nagoya City University Graduate School of Medical Sciences, Nagoya, Japan; Eiji Takasawa, Department of Orthopedic Surgery, Gunma University, Graduate School of Medicine, Gunma, Japan; Kazuki Takeda, Department of Orthopedic Surgery, Keio University School of Medicine, Tokyo, Japan and Department of Orthopedic Surgery, Japanese Red Cross Shizuoka Hospital, Shizuoka, Japan; Norihiko Takegami, Department of Orthopedic Surgery, Mie University Graduate School of Medicine, Mie, Japan; Koji Tamai, Department of Orthopedic Surgery, Osaka Metropolitan University Graduate School of Medicine, Osaka, Japan; Hidetomi Terai, Department of Orthopedic Surgery, Osaka Metropolitan University Graduate School of Medicine, Osaka, Japan; Hiroto Tokumoto, Department of Orthopedic Surgery, Graduate School of medical and Dental Sciences, Kagoshima University, Kagoshima, Japan; Hiroyuki Tominaga, Department of Orthopedic Surgery, Graduate School of medical and Dental Sciences, Kagoshima University, Kagoshima, Japan; Hitoshi Tonomura, Department of Orthopedics, Graduate School of Medical Science, Kyoto Prefectural University of Medicine, Kyoto, Japan; Tomohiro Yamada, Department of Orthopedic Surgery, Hamamatsu University School of Medicine, Shizuoka, Japan; Department of Orthopedic Surgery, Nagoya Kyoritsu Hospital, Aichi, Japan; Akihiro Yamaji, Department of Orthopedic Surgery, Ibaraki Seinan Medical Center Hospital, Ibaraki, Japan; Toshitaka Yoshii, Department of Orthopedic Surgery, Tokyo Medical and Dental University, Tokyo, Japan; Atsushi Yunde, Department of Orthopedic Surgery, Graduate school of Medicine, Chiba University, Chiba, Japan; Masashi Uehara, Department of Orthopedic Surgery, Shinshu University School of Medicine, Nagano, Japan; Hiroshi Uei, Department of Orthopedic Surgery, Nihon University School of Medicine, Tokyo, Japan and Department of Orthopedic Surgery, Nihon University Hospital, Tokyo, Japan.
